# Inhaled N, N-dimethyltryptamine diminishes connectivity between the ventral tegmental area and the nucleus accumbens: relevance to pathologies of mesolimbic and mesocortical pathways

**DOI:** 10.1038/s41598-025-31431-1

**Published:** 2025-12-12

**Authors:** Gisela Lima, Carla Soares, Marta Teixeira, Marta Pais, Célia Cabral, Patrícia Rijo, Miguel Castelo-Branco

**Affiliations:** 1 Coimbra Institute for Biomedical Imaging and Translational Research (CIBIT), University of Coimbra, Coimbra, Portugal; 2 Institute of Nuclear Sciences Applied to Health (ICNAS), Coimbra, Portugal; 3 Faculty of Medicine (FMUC), Institute of Physiology, University of Coimbra, Coimbra, Portugal; 4 Clinic Academic Center of Coimbra (CACC), Coimbra Institute for Clinical and Biomedical Research (iCBR), Faculty of Medicine, University of Coimbra, Coimbra, Portugal; 5 Center for Innovative Biomedicina and Biotechnology (CIBB), University of Coimbra, Coimbra, Portugal; 6 Department of Life Sciences, Centre for Functional Ecology, University of Coimbra, Coimbra, Portugal; 7 CBIOS-Universidade Lusófona’s Research Center for Biosciences & Health Technologies, Lisbon, Portugal

**Keywords:** Psychedelics, N,N-dimethyltryptamine, Reward, Dopamine, Mesolimbic, Resting-state, Neuroscience, Psychology, Psychology

## Abstract

Reward processing is a broad psychological construct that can be parsed into distinct components known as “reinforcement learning” (learning), “reward responsiveness” (liking), and “motivation to obtain a reward” (wanting). Dysfunctions in reward processing in mesolimbic and mesocortical pathways are a core feature of many pathologies. Psychedelics have been proposed as a treatment option for multiple disorders affecting the reward system, but mechanistic studies are lacking. In this preliminary, hypothesis-generating pharmacoimaging study, we evaluated the effects of inhaled N, N-dimethyltryptamine (DMT) with a particular focus on the connectivity of the mesocorticolimbic circuitry. Our within-subject pharmacoimaging design included 11 healthy participants with prior experience in psychedelics. In the active condition, DMT was self-administered immediately before MRI acquisition, while in the control condition there was no administration. We found decreased connectivity between the right nucleus accumbens (NAc) and the left ventral tegmental area (VTA), increased connectivity between the right NAc and anterior cingulate cortex (ACC) and increased connectivity between the medial prefrontal cortex (mPFC) and the ACC. These results correlated with changes in volition and perception, as measured with the hallucination rating scale. In sum, we found reduced connectivity in the midbrain-NAc pathway, which connectivity is often increased in addiction, and increased connectivity between reward/affective regions and the ACC. These findings suggest a potential therapeutic potential of psychedelics in disorders affecting reward processing.

## Introduction

The neural systems underlying reward are still largely debated. In general, it is accepted that reward has three main separable functions: liking or pleasure, wanting or the motivation to obtain pleasure, and learning, including prediction of future values, approach and decision making^1–3^. “Liking” refers to the hedonic impact from a pleasurable experience that can be objectively measured. “Wanting” refers to attribution of incentive salience to reward-related stimuli, causing both reward and cue to become motivationally desired and pursued^4^. Learning mechanisms allow the prediction of outcomes and behavioral adaptation, driven by reward prediction error models^5^.

The mesolimbic and mesocortical reward circuits are two of the brain’s major dopaminergic pathways, both originating primarily in the ventral tegmental area (VTA). The mesolimbic pathway involves bidirectional projections from the VTA to the nucleus accumbens (NAc) and other limbic regions, while the mesocortical pathway projects to the prefrontal cortex (PFC), including the medial PFC (mPFC) and the anterior cingulate cortex (ACC)^6,7^.

Neuroimaging studies evaluating reward functions usually utilize decision-making tasks, focusing on the different reward stages, namely the reward anticipation phase, the motivational phase and the consummatory phase or outcome. First, a reward draws attention and facilitates detection. Subsequent comparison with previous representations determines its novelty and saliency^8^. After the identification, a value for the reward is determined, driven by behavioral preferences. Value draws attention because it induces motivational salience. Stimulus-driven attention can be induced by various forms of salience, including physical, novelty, surprise and motivational, ultimately impacting selection of information and modulation of neuronal processes^9,10^. Learning processes such as Pavlovian and operant conditioning allow outcome anticipation based on past associations^11^. Reward learning is strongly shaped by reward prediction errors, where discrepancies between expected and actual outcomes drive the updating of value expectations and guide behavior^2^. When these learning signals become distorted, aberrant anticipations of reward or threat cues (aberrant salience) can emerge, contributing to maladaptive behaviors across psychopathological conditions^12^. Finally, consuming a pleasurable reward makes one want to go back for more while receiving a punishment makes avoidance actions more likely in the future. Actions, when repeated or overtrained, can become habits, which are automatic and inflexible responses^11^. Habits become independent responses from the outcome and are insensitive to reward devaluation and contingency degradation. Habits reach a pathological status when they become maladaptive and are very resistant to extinction when contingencies change^13^.

Dysfunctions in reward processing are expressed clinically as anhedonia, depressed mood, loss of pleasure, impulsivity, compulsivity, maladaptive habits and cognitive and behavioral inflexibility in multiple disorders^14,15^. In the same way, altered mesocorticolimbic activity patterns are also characteristic, for example of, depression, substance use disorders (to drugs, gambling and food), attention deficit hyperactivity disorder, obsessive–compulsive disorder, autism, schizophrenia and chronic pain^16–20^. Together, results support that a dysregulated mesocorticolimbic circuitry is a core find in various psychiatric disorders, and that the specific symptoms may be related to those aberrant activations and connections^21^.

Psychedelics have been shown to modulate mesocorticolimbic and associative circuits involved in reward and affective processing. For example, an electrophysiological study reported that low doses of lysergic acid diethylamide (LSD) enhanced reward-related potentials during the monetary incentive delay task in healthy adults, reflecting increased hedonic, motivational, and affective processing^22^. A recent meta-analysis of fMRI of tryptamine psychedelics (including LSD, psilocybin, DMT and ayahuasca) further demonstrated convergent alterations in mPFC, posterior and ACC, supramarginal gyrus and temporal regions, with a robust modulatory effect in the right amygdala^23^. These regions are central to reward valuation, salience attribution and affective regulation, supporting the view that psychedelics engage neural systems involved in motivation and emotion.

N, N-Dimethyltryptamine (DMT) is a tryptamine psychedelic found in nature that displays agonist activity in several serotonergic receptors, especially in the 5-HT1A, 5-HT2A, and 5-HT2C receptors^24^. Other receptors have been investigated as targets, namely glutamate receptors, dopamine, trace amine-associated (TAARs), sigma-1 and opioid receptors^25^. Despite DMT being inactive after oral intake, when inhaled, insufflated or given intravenously, it produces a very rapid and potent onset, with peak effects at 3–10 min and lasting 5 to 15 min^26^. The psychedelic effects are characterized by initial fear or anxiety responses, followed by excitation and positivity, detachment or dissociation from the body, changes in though content and intense visual imagery, as well as dose-dependent elevations in heart rate, mean arterial blood pressure and endocrine effects^27,28^.

Functional MRI studies have provided the first evidence of how DMT alters brain connectivity in humans. A placebo-controlled within-subject study with intravenous DMT (20 mg) in healthy participants, showed widespread increases in global functional connectivity, breakdown of within-network integrity across most canonical networks, and a compression of the unimodal-transmodal cortical gradient, suggesting a globally integrated brain state^29^. A multimodal fMRI-EKG study further identified a dynamic substate immediately after intravenous DMT, marked by hippocampal and medial parietal deactivations together with increased superior temporal activity^30^. Finally, a recent resting-state functional connectivity (rsFC) study with inhaled DMT reported increased functional connectivity between the supramarginal gyrus, posterior cingulate cortex, amygdala, and orbitofrontal cortex, highlighting modulation of socio-affective circuits^31^. Together, these findings suggest that DMT profoundly alters brain network dynamics at both global and regional levels. The observed changes implicate salience, frontoparietal, limbic-hippocampal, and socio-affective networks, which are tightly coupled to motivational salience, emotional learning and reward sensitivity.

Clinical and preclinical studies support the potential therapeutic role of DMT in disorders characterized by reward dysfunction. In treatment-resistant depression, both intravenous and vaporized DMT have been shown to rapidly reduce depressive symptoms, including anhedonia, with effects sustained up to one month^32,33^. Preclinical work in a chronic stress model also demonstrated that a single dose of DMT reversed anhedonia and cognitive deficits, outperforming fluoxetine^34^. Additional evidence for the therapeutic potential of DMT comes from studies with ayahuasca, a brew that contains DMT and a monoamine oxidase inhibitor, preventing first-pass effect at the digestive tract, thus allowing DMT´s absorption and systemic and psychoactive effects. Randomized controlled trials demonstrated rapid and sustained antidepressant and anxiolytic effects of ayahuasca in treatment-resistant depression and social anxiety disorder, as well as reductions in suicidality^35–38^. Beyond mood and anxiety, a systematic review of preclinical and human studies also points to beneficial effects in substance use disorders, where ayahuasca was associated with reduced self-administration, decreased substance use, and improved quality of life^39^. Emerging evidence further suggests potential benefits of ayahuasca in other conditions characterized by maladaptive reward and affective processing, including eating disorders, post-traumatic stress disorder and pain^40–42^.

Although the above evidence indicates a possible interaction between DMT and reward-related circuits, no study to date has directly examined the effects of inhaled DMT on rsFC within the mesocorticolimbic circuit in healthy humans. Studying healthy participants allows us to characterize the direct neural effects of DMT without the potential confounds of clinical populations, such as medication or disease-related neurobiological alterations. The primary aim of this preliminary study was to determine how inhaled DMT versus control affects rsFC between reward-related brain areas. Our hypothesis was that DMT would alter connectivity between key brain regions within the mesocorticolimbic dopamine system.

## Materials and methods

### Study design

This study used a within-subject design, consisting of an active and a control condition. The active condition consisted of self-administered (via inhalation) approximately 50 to 70 mg of DMT (root bark of *Mimosa hostilis* Benth., synonym of *Mimosa tenuiflora* (Willd.) Poir.,), immediately before MRI acquisition. In the control condition, no inhalation occurred. This 50–70 mg range was chosen to match established inhaled DMT doses (~ 40–100 mg) that reliably produce strong yet transient effects compatible with the fMRI acquisition timing^25^.

The quantification of DMT present in the sample was performed on high-performance liquid chromatography (HPLC-DAD) and revealed a presence of 30.92% DMT. For details on DMT quantification, please refer to Pais et al. (2024)^43^. Each individual dose was weighed just before administration.

The washout periods between sessions were at least one month, with a mean interval of 86 days (range 34–215 days). All participants underwent the DMT condition first, followed by the control session. This fixed order respected the ritualistic set and setting of administration and minimized expectancy effects that could arise if a neutral control was experienced beforehand and consequently bias reward-related responses.

Written informed consent for the study was given, which was conducted according to the Declaration of Helsinki and subsequent revisions. Ethical approval was obtained from the ethics committee of the Faculty of Medicine of the University of Coimbra.

### Participants

Eleven healthy subjects (mean age SD 37 ± 12.4 years), 4 females and 7 males with previous experience with inhaled DMT and other psychedelics were recruited through social media and word-of-mouth. All participants underwent an initial online screening and a posterior physical, cognitive and neuropsychiatric screening evaluation. We used the Mini-International Neuropsychiatric Interview (M.I.N.I.), a short structured diagnostic interview for DSM-IV and ICD-10 psychiatric disorders; the Mini Mental State Exam (MMSE) for cognitive impairment screening and the Graffar Scale - Portuguese version, which measures socioeconomic status^44–47^.

Physical examination and vital signs were also assessed. Complementary exams such as blood and urine analysis or electrocardiogram were required in an individual basis, when applicable. Exclusion criteria included psychiatric disease (personal or family history of schizophrenia, bipolar disorder, mania or hypomania, current or past addictions); major or unstable medical co-morbidities, psychiatric, antihypertensive or sympathomimetic medications, and pregnancy, suspected pregnancy or breastfeeding. Subjects with conditions known to involve an altered function of the reward system, such as eating disorders, obesity or chronic pain, were also excluded. Participants were asked to abstain from recreational or psychoactive drugs for two months prior to the study session and caffeine and tobacco for the preceding 24 h. For detailed participant assessment and medical monitoring please see Lima et al. (2024)^48^.

### Study procedures

After the initial screening, an email was sent to participants containing information about the study protocol, dietary recommendations, general safety measures, and logistics. A series of videos explaining the functional magnetic resonance imaging (fMRI) technique was also provided to promote a sense of familiarity with the MRI room and sound. Both sessions took place at the Institute of Nuclear Sciences Applied to Health. A room next to the MRI suite was decorated to be comfortable and supportive of the study context, with pillows and dim light, where participants could relax, meditate, chant songs and play musical instruments and perform other ritualistic practices. Participants self-administered DMT immediately outside the fMRI suite. They handed the pipe to facilitators after exhalation, walked a five-step distance to the scanner and were helped to lie down. Between smoking, preparing and monitoring the participant in the MRI machine and starting the scan, a mean period of 4 min was recorded. Effects were felt during inhalation, peaked at 2–3 min, gradually diminished and by the time the MRI acquisition ended, there were no reported effects. In the control condition, participants performed the same ritualistic practices before the fMRI scan, but without DMT inhalation.

The entire session was monitored by two psychologists and a medical doctor, and participants were discharged at the end of the day after medical evaluation.

Additionally, to study the acute psychedelic experience, we used the Hallucinogen Rating Scale (HRS)^28^. This assessment instrument is a questionnaire consisting of 100 items and employs a rating scale from 0 to 4. Its purpose is to evaluate the subjective effects of psychedelic substances across six categories: somaesthesia (somatic and interoceptive effects), affect (emotional responses), perception (visual, auditory, gustatory and olfactory alterations), cognition. (changes in thought processes), volition (capacity to interact with the experience and maintain a sense of self-control), and intensity (overall strength of the experience)^49^. The HRS was administered after the MRI procedure, when psychoactive effects were absent and participants able and willing to talk clearly and share their experience with the team members. They were instructed to respond taking in consideration what they had experienced during the acute effects.

### MRI acquisition

MRI data were acquired using a 3 T imaging system (MAGNETOM Prisma, Siemens Medical Solutions) with a 64-channel head-coil. Resting-state functional images were collected during a 7-minute session in which participants rested with their eyes closed, comprising a total of 210 volumes. The resting-state BOLD fMRI protocol included the following parameters: Repetition Time (TR) = 2000 ms; Echo Time (TE) = 20 ms; flip angle = 82 degrees; slices = 50 of thickness = 2,5 mm; field of view (FOV) = 195 mm × 195 mm; voxel size = 2.5 mm × 2.5 mm × 2.5 mm. Hight-resolution anatomical images were also obtained using a T1-weighted anatomical MRI data at a spatial resolution of 1 × 1 × 1mm3, with a TR of 2530ms, TE of 3.5ms, and a Flip Angle of 7 degrees. The acquisition parameters followed a previously published protocol^43^.

### Data analysis

Results included in this manuscript come from analyses performed using CONN (RRID: SCR_009550) release 20.b and SPM (RRID: SCR_007037) release 12.7771^50,51^. Functional and anatomical data were preprocessed using a flexible preprocessing pipeline including realignment with correction of susceptibility distortion interactions, slice timing correction, outlier detection, direct segmentation and MNI-space normalization, and smoothing^52^.

Functional data were realigned using SPM realign & unwarp procedure, where all scans were coregistered to a reference image (first scan of the first session) using a least squares approach and a 6 parameter (rigid body) transformation, and resampled using b-spline interpolation to correct for motion and magnetic susceptibility interactions^53,54^. Temporal misalignment between different slices of the functional data (acquired in interleaved Siemens order) was corrected following SPM slice-timing correction (STC) procedure, using sinc temporal interpolation to resample each slice BOLD timeseries to a common mid-acquisition time^55,56^.

Outlier scans (framewise displacement > 0.5 mm or global BOLD signal change > 3 standard deviations) were identified and removed for each voxel, subject, and condition, following the standard preprocessing and denoising pipeline implemented in the CONN toolbox, which integrates the using the Artifact Detection Tool (ART) for outlier identification^57,58^. Functional and anatomical data were normalized into standard MNI space, segmented into grey matter, white matter, and CSF tissue classes, and resampled to 2 mm isotropic voxels following a direct normalization procedure using SPM unified segmentation and normalization algorithm with the default IXI-549 tissue probability map template^58–61^. Additionally, functional data were smoothed using spatial convolution with a Gaussian kernel of 8 mm full width half maximum (FWHM).

In addition, functional data were denoised using a standard denoising pipeline including the regression of potential confounding effects characterized by white matter timeseries (10 CompCor noise components), CSF timeseries (5 CompCor noise components), motion parameters and their first order derivatives (12 factors), outlier scans (below 182 factors), and linear trends (2 factors) within each functional run, followed by high-pass frequency filtering of the BOLD timeseries above 0.01 Hz^62,63^. CompCor noise components within white matter and CSF were estimated by computing the average BOLD signal as well as the largest principal components orthogonal to the BOLD average, motion parameters, and outlier scans within each subject’s eroded segmentation masks^64,65^.

ROI-to-ROI connectivity matrices were estimated characterizing the patterns of functional connectivity with 8 ROIs which were selected based on previous reward studies: (i) bilateral ventral tegmental area (ii) bilateral nucleus accumbens (iii) bilateral amygdala (iv) anterior cingulate cortex (v) medial prefrontal cortex^66,67^. ROIs. Functional connectivity strength was quantified using Fisher-transformed bivariate correlation coefficients, derived from a weighted general linear model (weighted-GLM). These coefficients were computed separately for each pair of ROIs, based on the relationship between their BOLD signal timeseries. The contribution of each scan was modulated by a boxcar signal representing each experimental condition convolved with the canonical hemodynamic response function from SPM, and subsequently rectified.

At the Group-level, analyses were conducted using a General Linear Model (GLM) with random-effects modeling across participants. Statistical significance was assessed using an FDR-corrected threshold of p-FDR < 0.05 across all ROI-to-ROI pairs to control for multiple comparisons. We employed a between-conditions contrast (DMT > Control) to identify ROI pairs showing significant connectivity differences between conditions^68,69^.

We used SPSS (version 28.0.0.0) to analyze the self-reported data obtained through the Hallucinogen Rating Scale (HRS). Employing a repeated-measures ANOVA, we focused on comparing the effects of the DMT and Control conditions. Significance was established at *p* < 0.05. To explore the connection between imaging and self-reported experiences, we conducted Spearman’s correlation analysis. Initially, we calculated the change in connectivity (Δr) between the DMT and control conditions. Additionally, we investigated the difference in each scale of the Hallucinogen Rating Scale scores (ΔHRS) between both conditions. Finally, we performed Spearman’s correlation analysis between Δr and ΔHRS across conditions, keeping a significance level of *p* < 0.05.

Effect sizes for the ROI-to-ROI functional connectivity analyses were calculated as the difference in Fisher z-transformed correlation coefficients between DMT and control conditions (Δz), reflecting the magnitude of connectivity changes between ROIs, whereas effect sizes for the self-report questionnaire data were reported as partial η².

To evaluate statistical power, we conducted a power analysis using G*Power based on Cohen’s d effect sizes observed in our previous study using this dataset ((Soares et al., 2024; range: d = 1.26–1.72). For paired-samples t-tests (two-tailed, α = 0.05, *n* = 11), using the most conservative effect size (d = 1.26) yielded an achieved power of 96.3% (Type II error rate = 3.7%).^**31**^.

## Results

### Functional connectivity

**Fig. 1:**
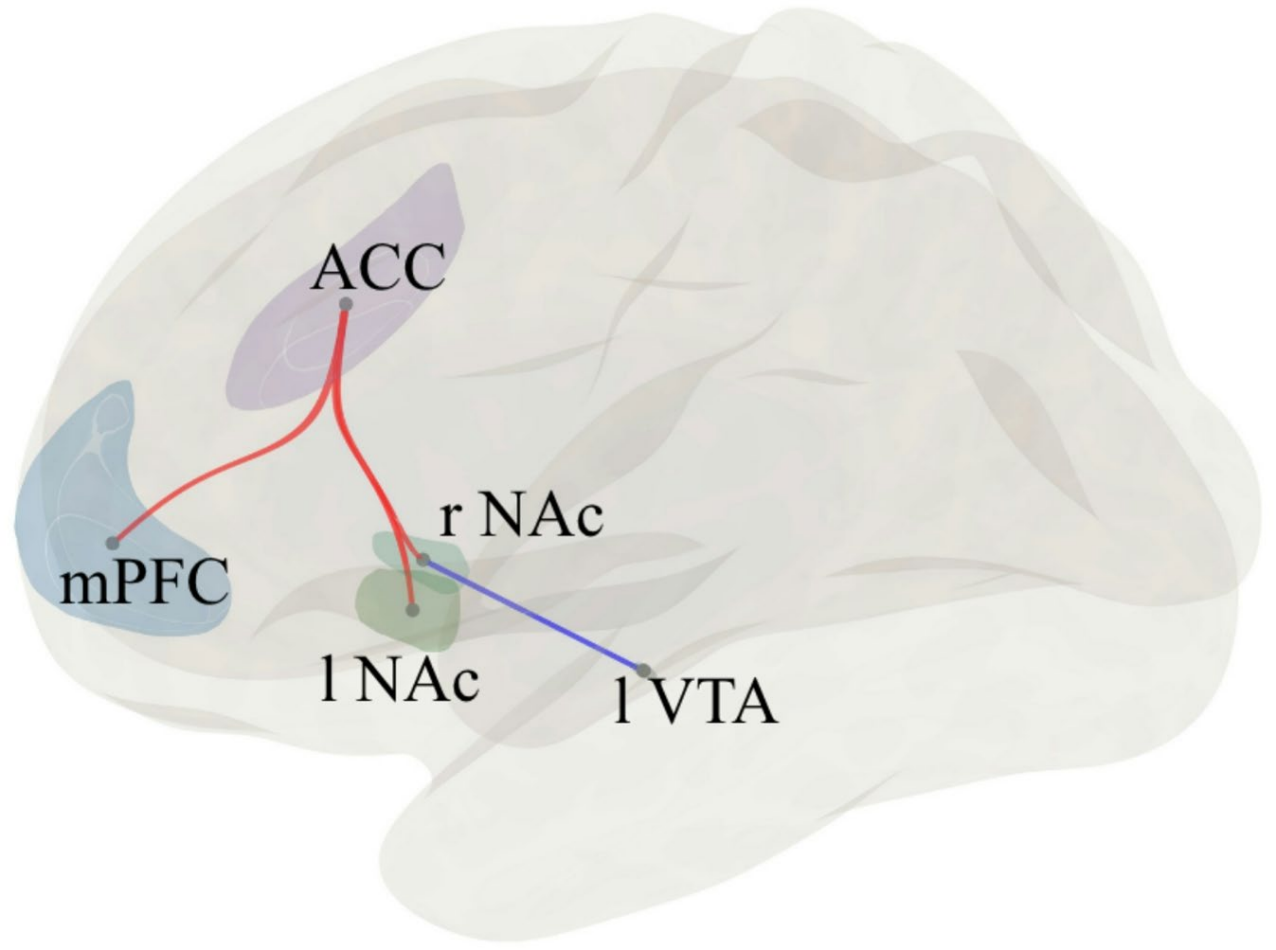
Illustration of ROI-to-ROI analysis results showing changes in rs-FC in the DMT condition compared to control. Left view of the brain with both hemispheres is displayed. The line colors represent t-statistics. Red lines connect ROIs with increased functional connectivity, while blue lines correspond to decreased connectivity. *ACC* Anterior Cingulate Cortex, *l NAc* left Nucleus Accumbens, *mPFC* medial Prefrontal Cortex, *r NAc* right Nucleus Accumbens, *l VTA* left Ventral Tegmental Area.

**Fig. 2:**
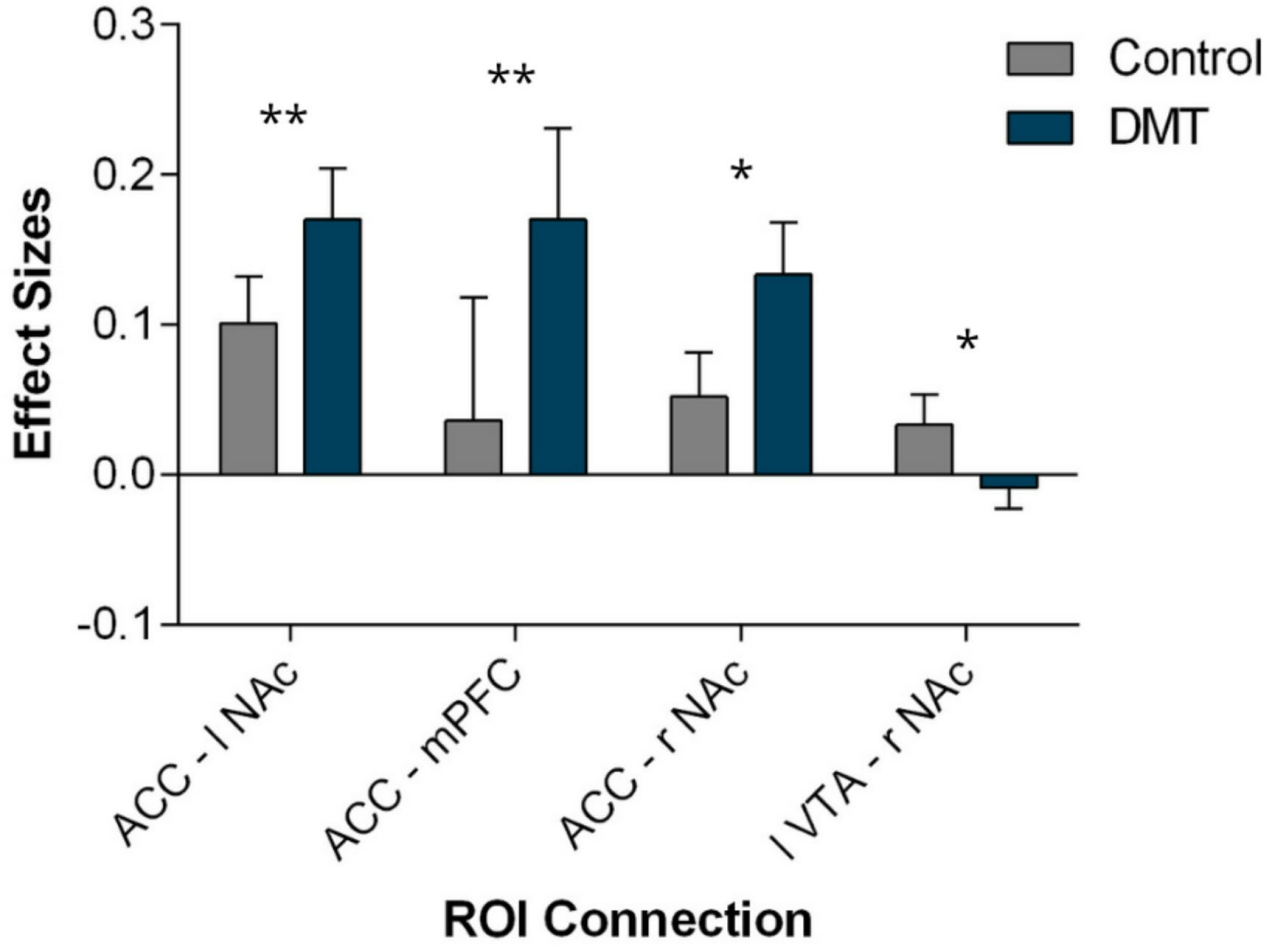
Functional connectivity between the pairs of ROIs showing statistically significant changes in the contrast DMT > Control, at a cluster level p-FDR corrected threshold of *p* < 0.05. Bars show mean scores, error bars show standard error of the mean. Effect sizes represent Fisher-transformed bivariate correlation coefficients for each pair of ROIs. Asterisks correspond to the following significance levels: *p < 0.05 and **p < 0.01.

DMT and control conditions were compared across subjects using a within subject design. Table 1 presents the participants’ sociodemographic characteristics. To investigate functional connectivity changes between the two conditions, we conducted an ROI-to-ROI correlation analysis using core regions of the reward system. The results revealed statistically significant changes in functional connectivity between DMT and control conditions for the mPFC, ACC, bilateral NAc and left VTA (F(1,10) = 20.99, *p* = 0.006, Δz = 0.061, FDR-corrected). Figure 1 illustrates the ROI connection changes, and Fig. 2 shows the respective effect sizes. We observed decreased rsFC between the right NAc and the left VTA (t(10)=−2.25, *p* = 0.048, Δz = − 0.042), along with increased rsFC between the right accumbens and the ACC (t(10) = 2.74, *p* = 0.021, Δz = 0.081), mPFC and ACC (t(10) = 3.45, *p* = 0.006, Δz = 0.134), and left NAc and the ACC (t(10) = 4.24, *p* = 0.002, Δz = 0.069). Additionally, a tendency towards an increase in functional connectivity between the amygdala and ACC was observed; however, this did not withstand correction for multiple comparisons.

To confirm that connectivity changes were specific to the reward system rather than reflecting widespread non-specific effects, we examined connectivity between motor cerebellar and reward regions. No significant connectivity changes were observed between motor cerebellum and any reward region (all p-FDR > 0.05).

**Table 1:** Sociodemographic characteristics (*N* = 11).

Category	Count
N (male/female)	11 (7/4)
Age (Mean, SD)	37 ± 12.4 years
Education	
Upper secondary	3
University degree	8
Marital Status	
Single	7
Married	3
Widowed	1
Graffard Socioeconomic Index	
Medium	3
Medium-high	8
Professional Status	
Student	2
Employed	5
Unemployed	3
Retired	1

### Acute subjective effects

We observed significant differences across all HRS scales in the DMT condition compared to the control. Specifically, there were significant increases in Perception (F(1,10) = 78.64, *p* < 0.001, η2 = 0.89), somaesthesia (F(1,10) = 28.03, *p* < 0.001, η2 = 0.74), affect (F(1,10) = 27.28, *p* < 0.001, η2 = 0.73), cognition (F(1,10) = 17.56, *p* = 0.002, η2 = 0.64), volition (F(1,10) = 6.07, *p* = 0.033, η2 = 0.38), and intensity effects (F(1,10) = 129.05, *p* < 0.001, η2 = 0.93).

**Fig. 3:**
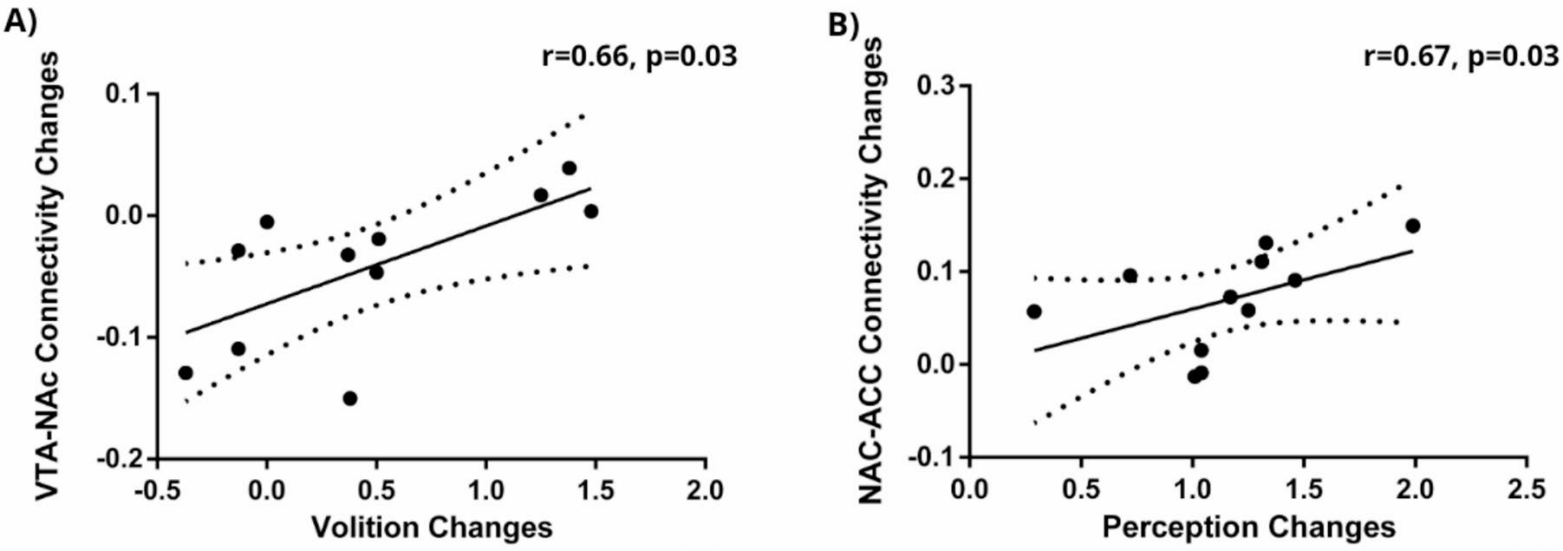
**A** Correlation between DMT-induced changes in VTA-NAc functional connectivity (Δr) and volition effects. **B** Correlation between DMT-induced changes in NAc-ACC functional connectivity (Δr) and perception effects. Lines represent linear fits with 95% confidence intervals (dotted lines). Spearman correlation statistics are reported (*N* = 11).

The correlation between the increases in these subjective effects and the DMT-induced changes in functional connectivity was analyzed using Spearman’s correlation. We observed a significant positive correlation between the changes in VTA-NAc functional connectivity and the increases in volition effects (r(9) = 0.66, *p* = 0.026), as well as between the changes in NAc-ACC connectivity and the increases in perception effects (r(9) = 0.67, *p* = 0.024). However, these associations did not survive FDR correction for multiple comparisons. These findings are depicted in Fig. 3.

No adverse events were reported or observed during or after the DMT and control sessions.

## Discussion

We explored rsFC alterations between reward related areas after inhaled DMT in healthy participants and found that inhaled DMT lead to decreased connectivity in mesolimbic core areas, namely between the right NAc and the left VTA and increased connectivity in mesocortical structures, specifically between the bilateral NAc and the ACC. We also found an increased connectivity between the mPFC and the ACC. The ACC has been identified has having a pivotal role in multiple processes stemming from the salience network (SN), and modulating the limbic system, reward pathways and pain neuromatrix^70^. Similarly, the mPFC is part of both the mesocortical reward system and the default mode network (DMN). In this way, our results suggest that DMT may simultaneously influence emotional, reward and executive functions.

DMT-induced functional changes between the VTA and NAc correlated with volition, which in the HRS refers to the subject’s capacity to interact with the experience and maintain a sense of willful self-control^49^. In our sample, higher increases in volition scores were associated with greater VTA-NAc connectivity changes, suggesting that modulation of this circuit may support self-referential awareness and agency during the acute psychedelic state. We also observed that connectivity changes between the NAc and ACC correlated with perceptual alterations, reflecting visual, auditory, gustatory and olfactory experiences. These findings suggest that the mesocorticolimbic changes induced by DMT are linked to interoceptive, self-referential, and perceptual processes.

The VTA and NAc are central hubs of the mesolimbic circuit, supporting motivational and affective functions. The VTA is crucial for detecting novel and salient stimuli and for signaling the motivational significance of events, with activity that can increase in response to both rewarding and aversive cues^71^. The NAc integrates VTA inputs with cortical signals to attribute value and salience to stimuli, shaping approach or avoidance behaviors^72^. In this context, our findings of reduced VTA-NAc connectivity under DMT may reflect a temporary dampening of incentive motivational signaling within this pathway. Such modulation could counteract the sensitization of “wanting” systems described in the incentive-sensitization theory of addiction, in which hyperreactivity of this circuit renders reward cues excessively salient and compulsively pursued^73,74^.

The ACC, by contrast, is a major integrative hub linking reward, emotion and executive control. It contributes to salience detection, affective regulation, and value-based decision-making, enabling adaptive behavioral responses^75,76^. The strengthened coupling between the NAc and ACC observed under DMT may therefore reflect enhanced integration of affective and motivational processes, contributing to a rebalancing between subcortical and cortical components of the reward circuitry. This interpretation aligns with previous evidence that psychedelic compounds modulate ACC activity and may rebalance salience and reward attribution^70^. Overall, the observed pattern of reduced VTA-NAc coupling and increased NAc-ACC connectivity, may indicate a reorganization of functional relationships within the mesocorticolimbic system, which could be related to the subjective effects of volition and perception and may help explain the hypothesized therapeutic potential of DMT in disorders of reward dysregulation.

While dopaminergic projections between the VTA and NAc have long been linked to reward and addiction, DMT acts on multiple neurotransmitter systems. Beyond its agonism at serotonergic receptors, DMT also interacts with glutamatergic, sigma-1, dopaminergic, TAAR and other receptor systems^77–79^. Although this pharmacological profile raises the possibility that the connectivity changes observed may reflect the combined modulation of several neurochemical pathways, serotonergic-dopaminergic interactions remain particularly relevant for motivational and reward processing, suggesting a plausible link to the observed modulation of mesocorticolimbic connectivity in our study.

Reward prediction errors represent the mismatch between expected and actual outcomes, serving as teaching signals in reinforcement learning^80^. Positive RPEs increase dopaminergic firing, reinforcing the association between cues and outcomes, while negative RPEs weaken such associations^2^. The magnitude of dopamine release scales with the prediction error magnitude, meaning that increasingly larger discrepancies are required to elicit equivalent reinforcement signals^81^. The VTA-NAc pathway is central to this process, providing the subcortical circuitry through which RPEs are generated and transmitted^82^. The ACC supports higher-order reinforcement related processes, including novelty detection, error monitoring, and action-outcome learning^83^. Novel and salient cues are compared with previous expectations and reward representations, assessing reward value and utility, giving rise or not to a prediction error. Taken together, our findings of reduced VTA-NAc connectivity and strengthened NAc-ACC coupling may reflect a temporary dampening of subcortical reinforcement signals, alongside enhanced cortical monitoring and evaluative functions. This dual pattern suggests that DMT may modulate both the generation of reinforcement learning signals and their cortical integration, with potential implications for how prediction errors are processed under psychedelic state.

In addition to reinforcement learning, the ACC is also central to emotional appraisal, motivation, and value-based decision-making^84^. The observed increase in NAc-ACC connectivity may reflect enhanced integration of motivational and affective processes during the acute DMT effects. This interpretation aligns with previous studies exploring acute regional cerebral blood flow produced by ayahuasca using SPECT, where acute administration increased regional activity in the right ACC, an effect linked to the subjective state of feeling one´s own bodily state and motivational aspects of emotion^85^.

Structural MRI studies have reported increased thickening of the ACC in long-term ayahuasca users, a finding that was related to attentional and cognitive control processes^86^. While the present results cannot establish causality, they provide preliminary evidence consistent with the hypothesis that DMT modulates cortico-striatal circuits involved in salience evaluation and emotion-cognition integration. Importantly, abnormalities in the connectivity between the NAc and the ACC have been consistently implicated in mood disorders such as depression, where reduced connectivity has been associated with both symptom and illness course^87^. Our findings of increased connectivity between these regions under DMT resonates with finding from clinical trials showing rapid antidepressant effects of ayahuasca and DMT^32,37^. Although our sample consisted of healthy participants, the connectivity pattern aligns with therapeutic findings, supporting the hypothesis that cortico-striatal modulation may underlie antidepressant effects of psychedelics.

Inhaled DMT has also been reported to produce long lasting analgesic effects^88^. Several analgesic mechanisms induced by psychedelics have been proposed, from molecular mechanisms to their neural correlates. Reward processing impairments have been hypothesized as a possible mechanism for the comorbidity of pain disorders, depression and addiction, and explain the high comorbidity among these conditions^89^. Pain and reward are conceptualized as opposing processes, yet they rely on overlapping neural substrates. Transition to chronic pain is associated with mesolimbic dysfunction, while altered connectivity between the NAc and cortical regions such as the prefrontal cortex and ACC is thought to mediate the emotional and motivational dimensions of pain^90,91^. Our findings of increased NAc-ACC connectivity under DMT aligns with theoretical models proposing that psychedelics influence circuits for reward, affect and pain, though the functional significance of this modulation requires further investigation.

Another finding of our study was the increased mPFC-ACC connectivity, consistent with previous psychedelic studies reporting enhanced internetwork connectivity between regions of the DMN and SN^92^. Having an overlapping role in the DMN and in the mesocorticolimbic circuit, the mPFC is implicated in a vast array of processes, including decision-making, working memory, stimulus discrimination, stress responses, and emotional and behavioral control, and is also associated with various neuropsychiatric disorders^21,71^. The observed increased connectivity between these areas may reflect enhanced communication between cognitive and affective networks, supporting the integration of motivational, cognitive and emotional processes.

Previous ayahuasca studies have also reported ACC modulation. For example, a study exploring the neurological basis of the “after-glow” effects after ayahuasca intake, revealed a post-acute increase in coupling between the PCC and a sub-region of the AAC, which was correlated to enhanced mindfulness capacities^93,94^. Increased SN-DMN coupling was linked with altered levels of affect on the HRS scale, reflecting emotional responses during the acute psychedelic session. Aberrant connectivity between the SN and the DMN has been found in obsessive-compulsive disorder and a decrease in connectivity in general anxiety as well as in anhedonia^95–98^. In this context, the increased mPFC-ACC connectivity we observed may align with hypotheses that psychedelics promote greater flexibility in psychological and cognitive processing. More specifically, by modulating cortico-cortical interactions between DMN and SN hubs, DMT may contribute to the characteristic changes in self-referential and affective processing observed under the psychedelic state.

The recruitment of a relatively small sample of size of individuals is a limitation, which is partially mitigated by the within-subject design. This sample size limits the statistical power and ability to control for confounding variables such as age and sex. Future studies with larger samples are needed to replicate these findings and examine their robustness when accounting for demographic factors. Participants were experienced DMT users, limiting generalization to naïve populations. Sessions were conducted in a non-blinded, ritualistic self-administration setting, which may introduce expectancy or context effects. Resting-state functional connectivity provides only indirect measures of neural dynamics, and the timing of MRI may not fully capture the dynamic evolution of DMT’s acute effects.

In summary, this preliminary and hypothesis-generating study provides initial insights into how inhaled DMT modulates functional connectivity within mesolimbic and mesocortical reward pathways. This exploratory work lays the groundwork for future mechanistic and interventional studies aimed at testing whether modulation of the VTA-NAc and ACC-centered circuits could have therapeutic relevance for disorders of reward dysregulation, such as addiction, pain or depression.

## Data Availability

The raw data supporting the conclusions of this article will be made available by the authors, without undue reservation.
